# Multifaceted circuit functions of adult-born neurons

**DOI:** 10.12688/f1000research.20642.1

**Published:** 2019-11-26

**Authors:** Cristina V. Dieni, Jose Carlos Gonzalez, Linda Overstreet-Wadiche

**Affiliations:** 1 Department of Ophthalmology and Visual Sciences, University of Alabama at Birmingham, Birmingham, AL, 35294, USA; 2 Department of Neurobiology, University of Alabama at Birmingham, Birmingham, AL, 35294, USA

**Keywords:** adult neurogenesis, dentate gyrus, circuit, excitability, inhibition, glutamate

## Abstract

The dentate gyrus continually produces new neurons throughout life. Behavioral studies in rodents and network models show that new neurons contribute to normal dentate functions, but there are many unanswered questions about how the relatively small population of new neurons alters network activity. Here we discuss experimental evidence that supports multiple cellular mechanisms by which adult-born neurons contribute to circuit function. Whereas past work focused on the unique intrinsic properties of young neurons, more recent studies also suggest that adult-born neurons alter the excitability of the mature neuronal population via unexpected circuit interactions.

## Introduction

The dentate gyrus (DG) is viewed as the main entry point for neural activity into the hippocampus, a brain region essential for learning and memory. The principal neurons, the dentate granule cells (GCs), are innervated by neurons of the entorhinal cortex that encode information about objects, spatial location, and details of the environment ^1– 3^. Thus, GCs integrate sensory and spatial information to help generate a neural representation of a context ^4, 5^. Interestingly, resident neural stem cells produce new GCs throughout adulthood, and these adult-born GCs receive synapses from cortical neurons via the perforant path and form output synapses with downstream neurons to participate in hippocampal network activity. Although the majority of neuroblasts undergo programmed cell death, surviving neurons are permanently incorporated into the DG and acquire properties similar to GCs generated early in development ^6– 8^. While adult-born GCs may replace a fraction of the developmentally generated population that is lost over time ^6, 9, 10^, their continuous addition accounts for the substantial increase in GC number across the first year of life in rodents ^11, 12^. How this process contributes to hippocampal learning and memory is an intriguing neurobiological question. Current insight into this question relies on experimentally tractable rodent models, but evidence of DG neurogenesis in healthy humans across the lifespan ^13, 14^ suggests that it could have implications for human cognition.

The DG has long been associated with the computational function of pattern separation or the transformation of afferent activity such that output patterns have less similarity than input patterns ^15, 16^. Brain regions involved in associative learning, such as the cerebellum and hippocampus, use circuits that perform pattern separation as a “pre-processing” step to help discriminate input patterns carrying sensory or contextual cues ^17^. Sparse population activity, wherein only a small fraction of principal neurons are active at a given time, is thought to be a critical component of pattern separation ^17^. In the DG, sparse activity is predicted to allow distinct GC ensembles to encode similar (but not identical) patterns of afferent activity, with a large capacity to transform overlapping patterns in the cortex into non-overlapping patterns in CA3 ^18, 19^. Interestingly, selective manipulations of adult-born GCs are sufficient to alter behavior paradigms thought to rely on DG pattern separation (reviewed in 20). Adult-born neurons likely contribute to DG functions by providing different information-processing capabilities compared to the existing mature population and by modifying the activity of mature GCs via circuit interactions. Here, we will review multiple cellular mechanisms by which adult-born neurons may contribute to DG network activity with a focus on circuit interactions.

## Functional maturation of adult-born GCs

**Figure 1.**
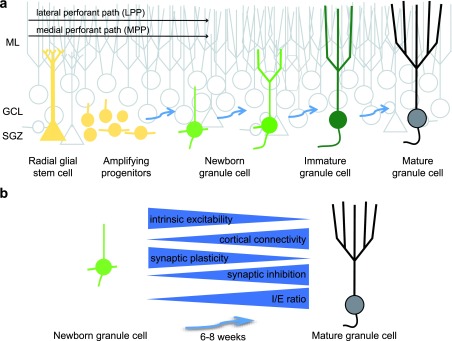
Functional maturation of adult-born granule cells. **a**. Illustration of the morphological maturation and integration of adult-born granule cells (GCs) (green cells) in the dentate gyrus. Progressive stages of integration are indicated by blue arrows. Yellow cells represent radial glial stem cells and proliferating progenitors. Developmentally generated GCs are gray and the molecular layer (ML), GC layer (GCL), and subgranular zone (SGZ) are indicated. GC dendrites extend through the ML and axons (truncated) project to CA3. **b**. Summary of the physiological changes that accompany morphological maturation of GCs. Full maturation typically requires 6 to 8 weeks in young adult mice. Blue arrows indicate direction of change, with intrinsic excitability and plasticity decreasing with maturation but synaptic connectivity from the cortex and synaptic inhibition increasing with maturation. The changes in adult-born GC intrinsic and synaptic properties during maturation are reviewed in 20– 23.

As newly born GCs develop and integrate into the DG ( Figure 1a), they exhibit dramatic changes in morphological, intrinsic, and synaptic properties that distinguish them from the larger population of developmentally generated GCs ( Figure 1b). Viral and transgenic tools have enabled detailed understanding of these changes in brain slice preparations (reviewed in 20– 23). In young adult rodents, mature electrophysiological properties typically develop over the course of 6–8 weeks, but there is variability in the rate of maturation depending on the age and species of the animal, housing conditions, and other factors ^24– 26^. Fully developed “mature” GCs share similar properties regardless of their birthdate and arise from a common progenitor population, consistent with the idea that adult neurogenesis represents a continuous extension of early development ^7, 27^.

Early studies converged on the idea that adult-born GCs transiently exhibit unique properties that provide novel information-processing capabilities to the mature circuit. For example, a high propensity for synaptic plasticity between 4 and 6 weeks after cell birth coupled with high intrinsic excitability affords an attractive explanation for the disproportionate contribution of a small population of young GCs within a sparsely active network ^28– 30^. Preferential recruitment by afferent stimulation suggests young GCs could act as integrators rather than discriminators of afferent activity patterns, raising the paradox that the addition of excitable young GCs could reduce rather than enhance pattern separation ^31, 32^. Thus, alternative computational functions for new neurons have also been proposed, including time encoding and memory resolution ^32, 33^. On the other hand, adult-born GCs might contribute to DG pattern separation primarily by modulating the excitability of the sparsely active mature GC population ^34– 36^.

Subsequent studies of the intrinsic properties of adult-born GCs continue to reveal how their shifting complement of ion channels and receptors can result in distinct information-processing capabilities compared to mature GCs ^37– 45^. One emerging idea is that despite the robust changes in various intrinsic and synaptic parameters during GC maturation ( Figure 1b), the net effect is a similar sparse afferent activation of all GC cohorts (reviewed in 23). For example, the high intrinsic excitability of young GCs is balanced by low innervation from cortical axons in a manner that potentially promotes sparse recruitment and discrimination of patterns due to limited sampling of input space ^42, 44^. *In vivo* analysis in behaving rodents combined with knowledge of circuit connectivity and activity patterns of afferent populations will be important to understand precisely how adult-born GCs are recruited during various behavioral states ^46– 49^. Yet distinct cell-autonomous properties may not fully explain the role of adult neurogenesis in DG circuit function, since developmentally generated GCs presumably mediate the majority of GC activity ^18, 47^. Rather, recent work has focused on understanding how adult-born GCs modify the activity of the pre-existing GC population via circuit interactions.

## Controlling mature GC excitability by feedback inhibition

Some of the first evidence that adult-born GCs alter the excitability of mature GCs came from voltage-sensitive dye imaging of hippocampal slices from mice with enhanced or ablated neurogenesis ^50^. In light of the high intrinsic excitability of young GCs, it was unexpected that the amplitude and spread of afferent-driven depolarization across the DG was reduced when neurogenesis was enhanced. Conversely, depolarization increased when neurogenesis was suppressed. The inverse relationship between the number of new GCs and excitability of the DG could result from adult-born GCs recruiting local gamma-aminobutyric acid (GABA)ergic interneurons that inhibit mature GCs, a circuit motif called feedback inhibition **.** Consistent with this idea, reducing neurogenesis with focal X-irradiation generated bursts of GC activity *in vivo* ^35^ and hypersensitivity to a chemoconvulsant ^51, 52^. While these studies assayed excitability across the population of GCs, subsequent work acutely silencing young GCs *in vivo* and *ex vivo* using designer receptors exclusively activated by designer drugs (DREADDs) also revealed enhanced excitability of individual mature GCs consistent with feedback inhibition ^53^.

**Figure 2.**
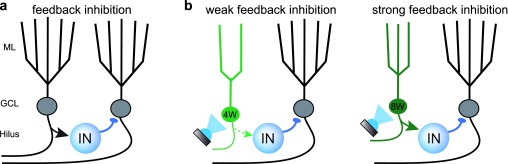
Controlling mature granule cell excitability by feedback inhibition. **a**. Depiction of feedback inhibition in the dentate gyrus (DG) wherein granule cells (GCs) recruit GABAergic interneurons (IN) that in turn inhibit GCs. Arrows indicate excitatory glutamatergic synapses and circles indicate inhibitory GABAergic synapses. A common feedback motif in the DG is lateral inhibition, where GCs inhibit their neighbors but not themselves ^57^. **b**. Summary of results from Temprana *et al*. ^58^ showing optogenetic activation of 4-week-old adult-born GCs (4W) generates little feedback inhibition (left) whereas activation of 8-week-old adult-born GCs (8W) generates effective feedback inhibition. The size of the arrow indicates the relative strength of interneuron recruitment. GCL, granule cell layer; ML, molecular layer.

In fact, GCs are expected to recruit interneurons in the hilus to generate feedback inhibition that contributes to sparse DG activity ^54– 56^ ( Figure 2a). Functional connectivity motifs for lateral inhibition, in which a GC innervates a GABAergic interneuron that innervates neighboring GCs but not themselves, are more abundant in the DG compared to other cortical regions ^57^. But whether young GCs exert a more powerful form of feedback inhibition compared to mature GCs, providing a unique contribution to sparse activity, was unclear. To address this question, Temprana and colleagues used a rigorous set of experiments to directly compare feedback inhibition from cohorts of GCs of differing ages expressing channelrhodopsin-2 (ChR2) ^58^. Whereas feedback inhibition generated by 8-week-old GCs suppressed perforant path-induced spiking, inhibition evoked by the same number of 4-week-old GCs had little effect ( Figure 2b). These results show that feedback inhibition produced by adult-born GCs increases with maturation and argues against the idea that 4-week-old GCs make a unique contribution to feedback inhibition. Similarly, other studies report that ablation of neurogenesis reduced dentate field excitatory postsynaptic potentials (fEPSPs) and population spikes *in vivo*, inconsistent with a prominent role for feedback inhibition in maintaining low excitability of the GC population ^35, 59^.

## Controlling mature GC excitability by glutamate-mediated excitation and inhibition

The plot thickened as subsequent work proposed that ChR2 activation of adult-born GCs younger than 7 weeks old provides strong inhibitory responses in neighboring GCs ^60^. These contrasting results could reflect a larger population of adult-born GCs that were manipulated or the use of repetitive rather than single stimuli. But, more remarkably, young GCs appeared to release both the inhibitory transmitter GABA and the excitatory transmitter glutamate directly onto mature GCs. Drew and colleagues ^60^ showed ChR2 activation of new GCs generated GABA _A_ and N-methyl-D-aspartate receptor (NMDAR)-mediated currents in mature GCs even when polysynaptic activity was blocked by α-amino-3-hydroxy-5-methyl-4-isoxazolepropionic acid receptor (AMPAR) antagonists. Such recurrent connectivity contrasts sharply with the conventional view that GCs provide a unidirectional glutamatergic projection, except in pathological conditions like epilepsy ^61, 62^. Yet the possibility that young GCs release both GABA and glutamate is consistent with a long-standing literature showing a transient dual-neurotransmitter phenotype, although the nature and prevalence of GABAergic signaling from young GCs has been controversial ^63– 65^.

**Figure 3.**
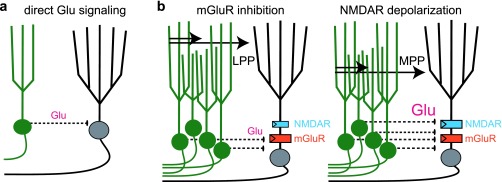
Controlling mature granule cell excitability by glutamate-mediated excitation and inhibition. **a**. Depiction of young granule cells (GCs) (green) releasing glutamate (Glu) directly onto mature GCs (black). **b**. Summary of the proposed circuit mechanism from Luna *et al*. ^66^, whereby direct glutamatergic signaling allows pathway-specific modulation of mature GC excitability. Left: stimulation of the lateral perforant path (LPP) recruits a small number of young GCs, leading to low Glu levels sufficient to activate extrasynaptic metabotropic Glu receptors (mGluRs) (orange) that hyperpolarize mature GCs. Right: stimulation of the medial perforant path (MPP) recruits a larger number of young GCs, releasing more Glu that also activates extrasynaptic N-methyl-D-aspartate receptors (NMDARs) (blue) that depolarize mature GCs.

Even as the efficacy of GABAergic feedback inhibition recruited by young GCs remains debated, a recent study further expands the potential repertoire of adult-born GC circuit functions by focusing on the direct glutamatergic signaling from young GCs to mature GCs ( Figure 3a). Luna and colleagues ^66^ first showed that silencing adult-born GCs increased mature GC responses evoked by stimulation of the lateral perforant path (LPP), whereas it reduced mature GC responses evoked by stimulation of the medial perforant path (MPP). Since the LPP carries sensory information and the MPP carries spatial information ^3^, adult-born GCs might selectively gate different forms of information during the creation of DG contextual representations in mature GCs. This unexpected pathway-specific regulation was attributed to activation of postsynaptic glutamate receptors on mature GCs rather than feedback inhibition. In fact, blocking GABA _A_ receptors did not block the responses evoked by new GCs, suggesting that feedback inhibition generated by young GCs is not a major circuit function in this paradigm.

Glutamate activates ionotropic AMPARs and NMDARs to depolarize neurons, but in some neurons, including GCs, glutamate also activates metabotropic glutamate receptors (mGluRs) coupled to potassium channels that cause hyperpolarization ^67, 68^. Luna and colleagues showed that optogenetic activation of young GCs evoked NMDAR-mediated excitatory postsynaptic potentials (EPSPs) in mature GCs as well as slow inhibitory postsynaptic potentials (IPSPs) driven by mGluRs. Such NMDAR- and mGluR-driven responses are characteristic of spillover signaling, wherein receptors located outside of synapses are activated by glutamate acting at a distance from the release site. Spillover transmission can occur in the absence of anatomically defined synaptic junctions or in addition to conventional point-to-point synaptic transmission ^68– 71^. The authors suggest that when a small number of young GCs are activated by LPP stimulation, the relatively low level of glutamate spillover favors mGluR-mediated inhibition, whereas a larger population of young GCs activated by MPP stimulation recruit stronger NMDAR-mediated depolarization that overrides mGluR inhibition. Thus, the number of young GCs recruited will affect their circuit function ( Figure 3b). In this manner, direct glutamatergic signaling from young GCs is poised to influence the information content of neural representations encoded by mature GCs.

## Modifying mature GC excitability by synaptic redistribution

Whereas Luna *et al*. ^66^ described circuit interactions that enable adult-born GCs to rapidly modify mature GC excitability in response to network activity, neurogenesis also changes synaptic structure and function of existing GCs over a longer time window as young GCs integrate into the circuit. The possibility that newborn GCs compete for synaptic input with existing GCs was implied by evidence that the survival of newborn GCs is competitively regulated by NMDAR activation ^72^. Subsequent anatomical analysis showed that immature dendritic spines transiently receive a high proportion of synapses from multiple-synapse boutons that could represent an intermediate structure in the transfer of cortical synapses from old to new GCs ^73, 74^ (but see 75). Whether the addition of adult-born GCs increases the number of synapses in the circuit or causes redistribution of existing synapses has important implications for understanding neurogenesis-induced plasticity. Indeed, changes in the number of GCs does not necessarily alter the number of input and output synapses. For example, early work showed that the density of middle molecular layer synapses is constant over the first year of postnatal life despite an increase in GC number ^11, 12, 76, 77^. In accordance with a redistribution of available presynaptic and postsynaptic elements, ablating GCs enhances the density of GABAergic somatic synapses on remaining GCs ^78^ and increasing the number of new GCs does not appear to alter the density of spines and synapses in the molecular layer ^79^. Recent support for the hypothesis that new GCs integrate into the circuit by competing for pre-existing synapses was provided by complementary studies showing that reducing the spine density of mature GCs enhanced the integration of new GCs ^80^ and that manipulating the number of newborn GCs inversely regulated synaptic connectivity of mature GCs ^81^. Thus, synaptic competition and subsequent redistribution of cortical connectivity may provide an additional mechanism for neurogenesis to contribute to sparsity and decorrelation of mature GC activity ^20^.

## Conclusions

The possibility that young GCs influence mature GC excitability by direct glutamatergic excitation and inhibition suggests an unexpectedly complex regulation layered upon additional secondary influences including feedback inhibition and synaptic competition. On the surface, it is difficult to envision the purpose of simultaneous direct excitation and inhibition. Luna *et al*. ^66^ propose that dual signaling allows young GCs to differentially regulate mature GC excitability depending on the strength of LPP versus MPP activity that differs between behavioral states and in the superior and inferior blade. Another possibility is that direct excitation/inhibition exerts local effects on neighboring GCs, whereas feedback influences GCs over a larger spatial domain as dictated by the extensive axonal arbors of GABAergic interneurons. Untangling the consequences of these multifaceted circuit interactions on hippocampal encoding and behavior will be an interesting challenge.

Many questions also remain about the mechanisms of these interactions at the cellular and synaptic level. For example, how is glutamate released from young GCs in a manner that generates receptor activation reminiscent of spillover signaling and where does this signaling occur? What is the efficiency of this novel signaling mechanism in controlling mature GC spiking, especially considering the stimulation of a single or small number of young GCs is insufficient to engage these interactions? How does the MPP recruit a larger population of young GCs than the LPP in light of evidence that the LPP preferentially innervates young GCs ^82^? Finally, what is the temporal relationship and relative efficacy of direct glutamatergic signaling in comparison with feedback inhibition and synaptic competition at each stage of new GC maturation? While the existing evidence indicates that the integration of new GCs has complex consequences for the local circuit, there is still a long way to go to understand how a few cells affect the many. The multi-layered complexity of the circuit interactions, in combination with activity-dependent regulation of adult neurogenesis, implies that continual neurogenesis provides extraordinary flexibility for adaptation to environmental changes and cognitive demands. Further exploring the cellular mechanisms by which adult-born GCs modify DG excitability and downstream hippocampal activity, along with cell-type-specific manipulations in behaving rodents to test their influence on behavior (reviewed in 83), will be important to decipher the full contribution of neurogenesis to memory encoding and retrieval.

## Abbreviations

AMPAR, α-amino-3-hydroxy-5-methyl-4-isoxazolepropionic acid receptor; ChR2, channelrhodopsin-2; DG, dentate gyrus; EPSP, excitatory postsynaptic potential; GABA, gamma-aminobutyric acid; GC, granule cell; IPSP, inhibitory postsynaptic potential; LPP, lateral perforant path; mGluR, metabotropic glutamate receptor; MPP, medial perforant path; NMDAR, N-methyl-D-aspartate receptor.
